# Botanical Description of British Plants

**Published:** 1808-06

**Authors:** 


### *Botanical Description of British Plants.*

[ Continued from our last, pp. 439—445. ]

Syngenesia. Frustanea.

36. Centaurea. *C. cyanus.*

*Ang.* Blue bottle. Knap weed. Corn flower. Hurt sickle. Batchelor's buttons.

*Gen. Desc.* Recept. bristly. Down either feathered or hair-like. Florets of the circumference funnel-shaped, irregular, longer than the others.

*Spec. Desc. Cal. scales outer,* serrated, green, cottony: *inner,* entire. *Leaves* strap-shaped, cottony, v. entire; *lower,* toothed. *Bloss.* bright blue, sometimes white, rose colour, or fine purple. *Filam.* surrounded just below the anthers with a fringe of silvery glandular hairs. *Anthers* almost almost black, horny at top. *Style* hairy beneath the summit. *Sum.* cloven. *Stem* 1 to 2 feet high, angular, firm, cottony, branched upwards. *Branches* 1 flowered. *Corn-fields.* Bl. June, August.

*Use.* The expressed juice of the petals is a good blue ink: it stains linen of a beautiful blue, but, in the mode in which it has hitherto been applied, the colour is not permanent. Mr. Boyle says that the juice of the central florets, with the addition of a very small quantity of alum, makes a lasting transparent blue, not inferior to ultramarine. *(See Gent. Mag.* 1748.*)* Cows, sheep, and goats eat it; horses and swine refuse it.—*Withering.*

Syngenesia. Necessaria.

37. Filago. *F. germanica.—Gnaphalium germanicum.*

*Ang.* Childing cudweed. Chafeweed.

*Gen. Desc.* Recept. naked. Down short, simple, sometimes none. Cal. tiled. Fem. flor. partly without petals, within the scales of the calyx.

*Spec. Desc. Panicle* forked. *Flowers* roundish, axillary, rough with hair. *Leaves* acute. See *gnaphalium germanicum No.* Bl. July, August.

*Use.* This plant has a drying and astringent quality; and the powder and decoction of it have sometimes succeeded in diarrhœas and dysenteries. It was formerly given by the English farmers to their cattle, in order to restore to them the faculty of chewing the cud; from whence it acquired the name of cudweed.—*Lightfoot.*

Class XX. Cryptogamia.

Cryptogamia. Miscellaneæ.

1. Equisetum. *E. arvense.*

*Ang.* Corn Horsetail.

*Gen. Desc.* Spike club-shaped, egg-oblong. Fructifications target-shaped, opening inwards.

*Spec. Desc. Fertile stalk* leafless. *Barren stem* leafy, lying down. *Leaves* in whirls, 12 to 12; joints of the upper branches frequently three-square, terminating in 3 open teeth. *Fertile stalks* appear first, soon decay. *Barren-st.* continue a long time, rough, slender, from 4 inches to 1 foot high, cylind. smooth, jointed, upper knots farther distant; *each joint* terminating in a wide, furrowed, many-cleft sheath. *Spike* yellowish, near 1 foot long. *Fructifications* in whirls, yellowish. *Root* cylind. with threads from the joints, stiffish, woolly, dark brown.

*Stalk*

*Stalk* fleshy, with 19 cylind. tubes within; 1 central, 9 round it, and 9 others v. minute between these and the central one.—*Moist cornfields.* Bl. March, April.

*Use.* It has a very strong astringent and diuretic quality, and is esteemed serviceable in hæmaturia and gonorrhœa, but it is abandoned by the present practice. If eaten by cows, an incurable diarrhœa is occasioned by it.—*Lightfoot.*

2. Equisetum. *E. hyemale.*

*Ang.* Rough horsetail. Shave grass. Pewter wort. Dutch rushes.

*Gen. Desc.* *As above.*

*Spec. Desc.* *Stem* naked, rough like a file, perennial, green, somewhat branched at the base, furrowed with 18 or 20 rough angles. *Joints* frequently 3 inches asunder. *Sheaths* pale, black at the base and edges, with 18 or 20 blunt teeth. *Spike* terminating.—*Marshy places, not common.* Bl. July, August.

*Use.* It is much used by turners, cabinet-makers, &c. for smoothing their works in wood, ivory, &c.; they call it shave-grass, and it is so hard as to take effect even upon brass and iron.—*Hill.* It is wholesome to horses, hurtful to cows, and disagreeable to sheep.—*Withering.*

3. Lycopodium. *L. clavatum.*

*Ang.* Common club moss. Wolf's claw.

*Gen. Desc.* Capsules axillary, kidney-shaped, two-valved, elastic; many seeded.

*Spec. Desc.* *Leaves* scattered, terminating in threads, strap-spear-shaped, pointed, and hooked. *Spikes* cylind. on fruit-st. in pairs, closely tiled with scales or husks, egg-spear-shaped, pointed, ragged: each scale encloses a kidney-shaped yellow capsule, exploding, when ripe, a yellow powder, which resembles sulphur, and burns with an explosion. *Stem* creeping; *shoots* several feet long, attached to the earth by strong woody fibres. *Branches* expanding, distant, trailing. *Fruit-st.* mostly with 2 equal spikes.—*Dry places, on mountains, heaths, woods.* Bl. July, August.

*Use.* In Russia and other countries, the powder of the capsules is used in medicine to heal galls in children, chaps in the skin and other sores: it is used also to powder over officinal pills. In Poland a decoction of this plant is used for the Plica polonica; a linen cloth being dipped in it and applied to the head, which is said to be cured by this fomentation.—*Lightfoot.* The seeds flash when thrown into into a flame; and are used, it is said, in the Russian and Swedish theatres to imitate lightening. Of this plant, in Sweden, door mats or basses are made. It restores ropy wine in a few days. The seeds are with difficulty made wet, and if they are scattered upon a bason of water, the hand may be dipped to the bottom without being wetted. *Withering.*

4. LYCOPODIUM. *L. selago.*

*Ang.* Fir-leaved club moss.

*Gen. Desc.* *As above.*

*Spec. Desc.* *Leaves* scattered, pointing 8 ways, spear-shaped, sharp pointed, stiff, smooth, shining, scolloped, cartilaginous at the edge. *Stem* upright, forked, 3 to 7 inches high, stiff; *branches* forked, closely covered with leaves, all of the same height. *Flowers* scattered. *Caps.* in the bosom of the upper leaves, kidney-shaped, flatted, yellow, opening like an oyster, pouring out a yellow powder, which is the seed. *Roots* forked like the stem.—*Mountainous heaths, clefts of rocks.* BL. April, Oct.

*Use.* This plant is cathartic and emetic, and destroys worms.—*Withering.* The Highlanders in Scotland make use of an infusion of it for the above purposes, but it is violent in its operation, and unless taken in a small dose, brings on convulsion and giddiness.—*Lightfoot.* The Swedes use a decoction of it to destroy lice on swine and other animals.—*Linn.* In the island of Ramsay, near Sky, this plant is made to answer the purposes of alum, in fixing colours used in dying.—*Lightfoot.* Its properties seem to challenge further inquiry.—*Withering.*

Cryptogamia. Filices.

5. OPHIOGLOSSUM. *O. vulgatum.*

*Ang.* Common Adder's tongue.

*Gen. Desc.* Caps. numerous, nearly globular, without an elastic ring; united by a membrane into a two-rowed spike; opening cross ways when ripe. Seeds numerous, minute.

*Spec. Desc.* *Leaf* egg-shaped, veinless, bearing the spike, embracing the stem, sometimes slightly lobed with appendages on one or both sides. *Spike* strap-shaped, green; when ripe, brown. *Stem,* solitary.—*Moist cold meadows.* BL. May, June.

*Var.* Fruit-st. *divided, each branch supporting a spike; sp. sometimes divided.*

*Use.* The common people use an ointment made of the fresh leaves as a vulnerary to green wounds: it is a very ancient application, having been recommended by Matthiolus, Tragus, &c.—*Lightfoot.*

6. Osmunda. *O. regalis.*

*Ang.* Osmund royal. Flowering fern. Royal moonwort.

*Gen. Desc.* Spike branched. Capsules distinct, sitting, globular, two-valved; without an elastic ring, opening either vertically or horizontally.

*Spec. Desc.* *Leaf* bearing the fructifications; doubly winged: *leafits* strap-spear-shaped, blunt, indistinctly serrated; *lower and younger* often lobed at the base; *upper wings* change into clusters of capsules and lose all appearance of foliage. *Fruct.* when ripe, red brown. *Caps.* open vertically. *Plant* 2 to 4 feet high, a pleasant transparent green.—*Watery places, boggy marshes.* Bl. July, August.

*Use.* The root boiled in water is very slimy; and in the North of Europe, is used instead of starch, to stiffen linen. Impressions of the leaves are frequent in the nodules of iron stone found in Coalbrook Dale iron-works. Dr. Withering says it is the only species of an indigenous vegetable that he has ever seen in a fossil state: but it is also a native of Virginia: all the other impressions of Filices which he has seen on iron stone, seem, he says, to be those of American plants.—*Withering.*

7. Pteris. *P. aquilina.*

*Ang.* Female Fern. Common Brakes.

*Gen. Desc.* Caps. disposed in a line under the reflected edge of the leaf.

*Spec. Desc.* *Leaves* more than doubly compound; *leafits* winged; *wings* spear-shaped; the lowermost wing cleft, the upper smaller. The *root* cut obliquely presents a representation of the Imperial Eagle, whence Linnæus has named it *P. Aquilina,* or eagle-brakes.—*Heaths and woods.* Bl. August.

*Use.* The ancients used this fern and the whole plant in diet drinks and decoctions, in chronic disorders of all kinds arising from obstructions of the viscera and spleen. The powder of it is given by the country people for worms; and it is imagined by them that a bed of the green leaves is a sovereign cure for the rickets in children. The poor in Normandy have sometimes made bread of the roots: and in Siberia they are frequently used in brewing ale, one part being mixed with two parts of malt. Fern is so astringent as, in many places abroad, to be employed for dressing kid and chamois leather. Fern, cut while it is green, and and left to rot on the land, is very good manure; and, if buried beneath potatoes, it never fails to produce a good crop.—*Lightfoot.* In many parts of England the common people mix the ashes of fern with water, and form the mixture into balls, which are afterwards made hot in the fire, and then used to make lye for scouring linen: from the ashes a tolerably pure alkali is obtained. It makes very durable thatch; and is an excellent litter for horses and cows. Where coal is scarce, it is used for heating ovens and for burning limestone; for it affords a very violent heat.—*Withering.*

8. ASPLENIUM. *A. scolopendium.—Lingua cervina. Hemionitis. Phyllitis.*

*Ang.* Spleen wort. Hart's-tongue.

*Gen. Desc.* Caps. disposed in straight and nearly parallel lines on the under surface of the leaf.

*Spec. Desc. Leaf* simple, heart-tongue-shaped, v. entire, 8 to 12 inches long, 1½ to 2½ inches broad. *Stalks* hairy. *Leaf-st.* rising from the root about 2 inches long. *Fruct.* in lines, slanting upwards from the mid. rib, but not in contact with it.—*Moist shady rocks, mouths of wells, old walls, mountain-tops.* BL. August, Sept.

*Var.* The leaves *curled or jagged, or many-cleft at the end.*

*Use.* It has an astringent quality. It is recommended to be taken, boiled in red wine, for hæmoptoe, diarrhœa, and dysentery. By the country people it is often used in the way of ointment as a vulnerary for burns and scaldings. *Lightfoot.* It is supposed to possess medicinal qualities in common with several other species of the same genus.—*Woodville.* See the following.

9. ASPLENIUM. *A. trichomanes.—Trichomanes mas. Polytrichum offic.*

*Ang.* Spleenwort. Milkwaste. Maiden-hair.

*Gen. Desc. As above.*

*Spec. Desc. Leaves* winged. *Leafits* either circular or oblong, scolloped; sometimes rather cut into lobes: *caps.* when ripe covering the whole under surface. *Plant* 3 to 7 inches high, consisting of a leaf, several of which rise singly from a black fibrous root, ¼ to ¾ inch broad.—*Old walls, rocks, shady stony places.* BL. May, Oct.

*Use.* The leaves have a mucilaginous, sweetish, subastringent taste. They are esteemed useful in disorders of the breast, proceeding from a thickness and acrimony of the juices; and are likewise supposed to promote expectoration toration of tough phlegm; and to open obstructions of the viscera. They are usually directed in effusion or decoction, with the addition of a little liquorice. A syrup prepared from them, though it has now no place in our Pharmacopœias, is frequently to be met with in our shops, both as prepared at home and imported from abroad. A little of these syrups, mixed with water, makes a very pleasant draught: the syrup brought from abroad has an admixture of orange flower water.—*Woodville.*

10. ASPLENIUM. *A. ruta-muraria.*

*Ang.* White maiden-hair. Wall-rue. Tent-wort. Rue maiden-hair.

*Gen. Desc.* *As above.*

*Spec. Desc.* *Leaves* doubly compound; divisions alternate. *Leafits* wedge-shaped, finely scolloped. *Stem* bare for near half its length. *Fruct.* in 2 or 3 rows, on each side the rib of the leaf.—*Old walls, moist crevices of rocks.* Fl. June, Oct.

*Use.* This was formerly received in the shops as a pectoral and deobstruent; and was recommended in coughs, asthmas, obstructions of the liver and spleen, and in scorbutic complaints; but it is now out of repute.—*Lightfoot.* This, and four other species of the same genus, were termed the five capillary herbs, and were formerly held in great estimation. To the taste they are slightly astringent, mucilaginous, and sweetish: they change the solution of iron to a black colour. They have been formerly used to strengthen the viscera, restrain hæmorrhages and alvine fluxes, expel gravel, and open the obstructions of the spleen and liver; as well as for the general purposes of demulcents and pectorals, (noticed before under the head of A. trichomanes.)—*Woodville.*

( To be continued. )

